# Cross-checking to reduce adverse events resulting from medical errors in the emergency department: study protocol of the CHARMED cluster randomized study

**DOI:** 10.1186/s12873-015-0046-1

**Published:** 2015-09-04

**Authors:** Yonathan Freund, Alexandra Rousseau, Laurence Berard, Helene Goulet, Patrick Ray, Benjamin Bloom, Tabassome Simon, Bruno Riou

**Affiliations:** Paris Sorbonne Université, UPMC univ-Paris 6, Paris, France; Emergency Department, Hopital Pitie-Salpetriere, Assistance Publique-Hôpitaux de Paris (APHP), Paris, France; APHP, GH HUEP, Hôpital St Antoine, Plateforme de recherche Clinique de l’est Parisien (URCEST-CRCEST), Paris, France; Emergency Department, Hopital Tenon, APHP, Paris, France; Emergency Department, Royal London Hospital, Barts Health NHS Trust, London, UK

## Abstract

**Background:**

Medical errors and preventable adverse events are a major cause of concern, especially in the emergency department (ED) where its prevalence has been reported to be roughly of 5–10 % of visits. Due to a short length of stay, emergency patients are often managed by a sole physician – in contrast with other specialties where they can benefit from multiples handover, ward rounds and staff meetings. As some studies report that the rate and severity of errors may decrease when there is more than one physician involved in the management in different settings, we sought to assess the impact of regular systematic cross-checkings between physicians in the ED.

**Design:**

The CHARMED (Cross-checking to reduce adverse events resulting from medical errors in the emergency department) study is a multicenter cluster randomized study that aim to evaluate the reduction of the rate of severe medical errors with implementation of systematic cross checkings between emergency physician, compared to a control period with usual care. This study will evaluate the effect of this intervention on the rate of severe medical errors (i.e. preventable adverse events or near miss) using a previously described two-level chart abstraction. We made the hypothesis that implementing frequent and systematic cross checking will reduce the rate of severe medical errors from 10 to 6 % - 1584 patients will be included, 140 for each period in each center.

**Discussion:**

The CHARMED study will be the largest study that analyse unselected ED charts for medical errors. This could provide evidence that frequent systematic cross-checking will reduce the incidence of severe medical errors.

**Trial registration:**

Clinical Trials, NCT02356926

**Electronic supplementary material:**

The online version of this article (doi:10.1186/s12873-015-0046-1) contains supplementary material, which is available to authorized users.

## Background

Medical errors are a major cause of morbidity and mortality and have been a topic of serious concern [1] since “To err is human” was published by the Institute of Medicine 1999. In the United States, medical errors are thought to be responsible of 100,000 deaths per year and more than one million injuries [1]. In France, 10 000 deaths and up to 3 % of all hospitalizations may be associated with medical errors [2]. For more than a decade, the rate of harm caused by medical errors has remained constant [3], although it is thought that more than a third of them could have been avoided [2, 4].

Emergency Departments (EDs) are busy places, where rapid decisions are made on the basis of incomplete information. Simultaneous management of multiple complex patients and lack of continuity of care can increase the likelihood of medical errors. Conditions in EDs replicate these risks for making medical errors and may amplify their consequences. Rising ED attendance rates in western countries, and subsequent ED overcrowding compounds the situation and further increases the risk of medical errors [5–8]. For these reasons, EDs are considered one of the most high risk environments for adverse events (AE) and serious AE resulting from medical errors. There are sparse data on the rate and severity of AE in the ED. Most previous studies have included passive or self-reporting method for error detection, which is associated with an underestimation of harm and frequency of medical errors. Reported rates of medical errors in the ED vary from 18 [9] to 32 % [10].

Recently, a large prospective study reported that severe medical errors (with the potential to provoke harm) occurred in 10 % of visits in the ED [4] in the US. In France, we conducted a preliminary study, for ED patients that were subsequently admitted, that corroborated these findings, with a medical error rate of 42 %, and an AE rate of 10 % [11].

### Study rationale

Due to the patient’s short length of stay, ED physicians often make independent management decisions. This is in contrast to other specialties, which may benefit from ward rounds, staff meetings, and handover. In our previous study [11], the single protective factor we found, i.e reducing the risk of AE, was the participation of more than one physician in the ED management. This included either the involvement of a resident or trainee in the patient care in addition to the senior physician, or a handover of the patient case in the ED. Recently, Kajdacsy-Balla Amaral et al. reported that night time cross coverage in intensive care unit (ICU) was associated with a significant decrease in mortality [12], with an odds ratio of 0.77 per 1 day of cross coverage. Interestingly, these findings are consistent with high-risk industrial settings, such as aviation setting, where every important decision, calculation or action needs to be cross checked by a peer. These high risk industries have a global mortality rate less than 1 per 100,000.

All these results question the time-honoured paradigm that associates handover and the involvement of more than one decision maker in the process of care with worse outcomes. To explain this, we aim to evaluate the influence of crosschecking physician decision and management in the ED with a peer. Our hypothesis is that the implementation of a systematic and frequent crosschecking within the ED between colleagues decreases the rate of medical errors and AE.

Cross checking may be a rapid and easy intervention to implement even in an overcrowded ED. Thus, this study will also report the feasibility of the implementation of systematic Cross Checking in the ED. We intend to include large numbers of centres in France, that treat diverse patient populations. Consequently, if our hypothesis is confirmed, this will confer a strong argument for future generalization of our intervention .

## Methods

### Study design

**Fig. 1:**
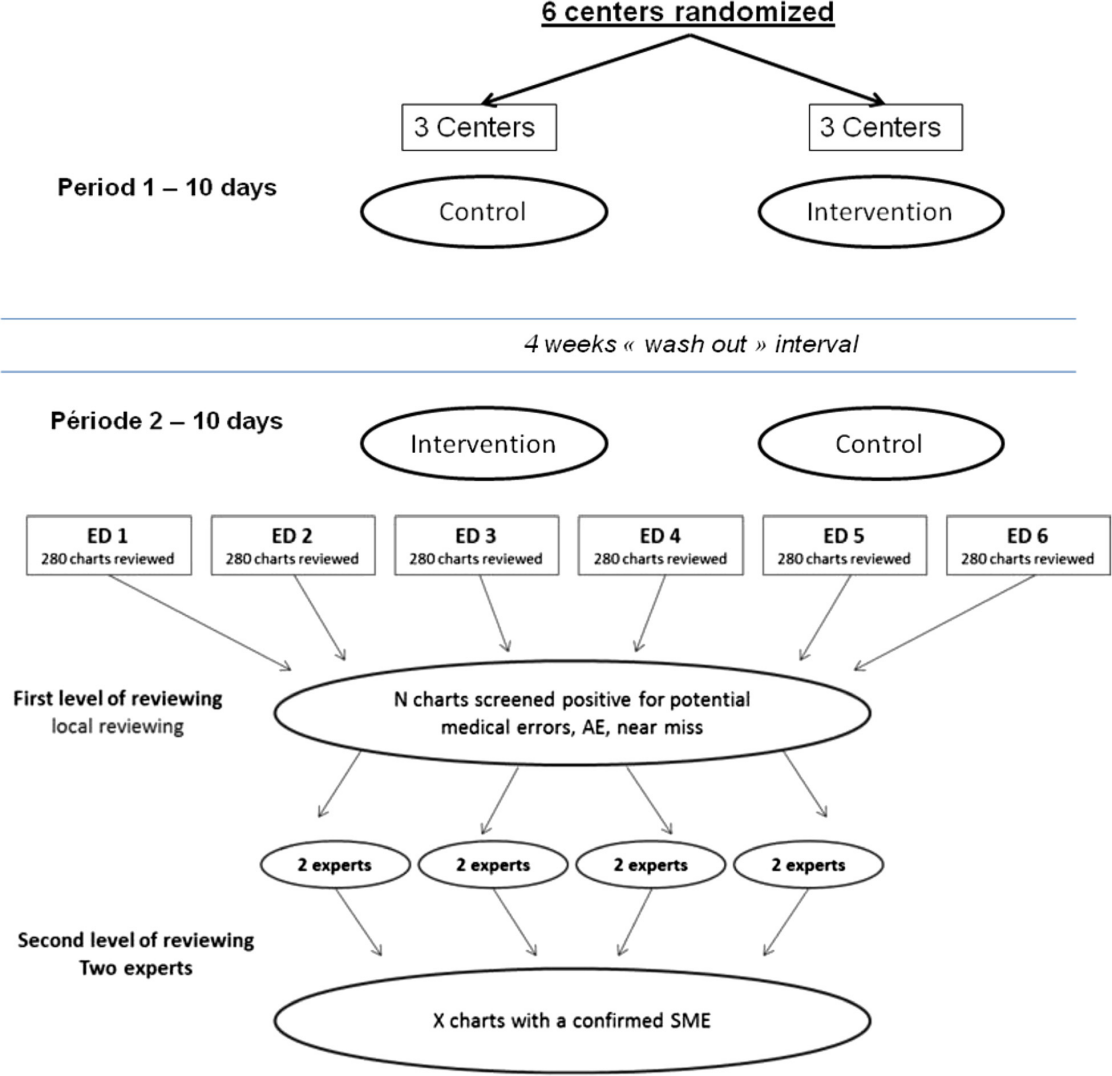
CHARMED study design for period allocation and detection of SME. ED: Emergency Department, SME: severe medical errors, AE: adverse event

The CHARMED study is a prospective, multicenter, cluster-randomized cross-over study in six EDs in France (NCT02356926). Centers will be randomly assigned to use routine management or systematic cross checking in the first period, and will use the alternative strategy in the second period (Fig. 1). Patients will be recruited in six centers in France. Our institutional review board (Comité de protection des personnes - Paris Ile de France 6) authorized the study without the need of signed informed consent as the study.

### Selection of patients

All patients that visit the ED during one of the two periods of recruitment, Monday to Friday between 8:30 am and 4:30 pm, will be screened for inclusion. We chose this time interval as they correspond to period that can be exposed to a cross checking, which will occur at 11:30 am, 2:00 pm and 4:30 pm. Patients with the following will be excluded:

1. Patients whose care is not provided by an EP (for example psychiatrist or maxillo-facial surgeon)
2. Scheduled return attendance to the ED
3. Low severity, defined by
  - Triage level 5 on a 1 to 5 scale (5 being the less severe) [13, 14]
  - Patients referred to a “minor” or “fast track” unit
  - Patients discharged home less than 1 h after first contact with an EP

After the completion of the two study periods, a clinician research technician (CRT) will electronically retrieve the list of patients that attended the ED during the recruiting period. Each center has electronic software that records all admission with the time of arrival and time of discharge. All patients that entered or left the ED within the recruitment period (i.e. Monday to Friday, 9 am to 5 pm, over a specified 2 week period) will be retrieved by their semi-anonymised number, hospital identification number), and the CRT will verify that they have no exclusion criteria. The independent methodology and research department will then randomly select 14 attendances for each of the 10 days of enrollment per period, for a total of 280 visits in each centre.

### Definition of key term and endpoints

In accordance to national and international recommendations [1, 2, 15], the following definitions will be used:

- **Medical Error**: Failure of a planned action to be completed as intended, or the use of a wrong plan to achieve an aim. The severity of an error will be classified using the National Coordinating Council on Medical Error and Reporting (NCCMERP) from A to I [16], as shown in Table 1.
- **Adverse event (AE)**: An injury that might have resulted from medical care (or lack thereof).
- **Near Miss**: A medical error that has the potential to cause an adverse event, but did not either by chance or after an intervention. A near miss is an error of severity B, C or D.
- **Preventable AE**: An AE associated with an error. A preventable AE is a medical error of severity E, F, G, H or I.
- **Severe Medical Error (SME)**: preventable AE or a near miss.

**Table 1:** National Coordinating Council for Medication Errors Reporting and Prevention (NCCMERP) classification of severity of medical errors

A	Circumstances or events that have the capacity to cause error
B	An error occurred but the error did not reach the patient
C	An error occurred that reached the patient but did not cause patient harm
D	An error occurred that reached the patient and required monitoring to confirm that it resulted in no harm and/or required intervention to preclude harm
E	An error occurred that may have contributed to or resulted in temporary harm
F	An error occurred that may have contributed to or resulted in temporary harm and required initial or prolonged hospitalization
G	An error occurred that may have contributed to or resulted in permanent patient harm
H	An error occurred that required intervention necessary to sustain life
I	An error occurred that may have contributed to or resulted in the patient’s death

The primary objective is to assess whether the implementation of Systematic Cross Checking in the ED will reduce the rate of severe medical errors. The primary endpoint is the rate of SME in the seven days following ED visits. Serious guidelines violation (local or national), even in the absence of any documented injury, will be considered as adverse events - As previously described, the subsequent adverse events might not clearly appear in the ED settings, hence its classification as a SME [8]. Hospital or ED re-attendance within the next 7 days will be also considered as an adverse event.

Secondary endpoints include the followings:

- Rate of AE and preventable AE
- Rate of Near Miss
- Severity of SME
- Factors associated to SME:
  - o Related to patient (age, chief complaint, comorbidities, triage level)
  - o Related to physician (grade, experience, number of physician involved, handoff)
  - o Related to the ED visit (Time of visit, daily occupancy, crowding, waiting time, length of stay, total number of emergency physicians)

### Experimental plan

In both periods, from 9am to 5pm, a CRT will be present in the ED to collect variables on providers and patients.

**In the control period**, usual care and routine management will be provided.

**In the intervention group**, systematic cross-checking will be implemented three times a day from 8:30 am to 6:00 pm between emergency physicians. The CRT will seek emergency physicians (EP) by pairs for crosschecking. Senior physicians will use peer crosschecking (i.e. crosschecker will also be an emergency senior physician). The CRT will assist the pairing. Each EP will present all his or her current patients. Patient presentation will be semi protocolised (see below), although usual presentation will be sought as this is the presentation method already in place for handover. The crosschecking will occur in the presence of the CRT and in the ED, in any medical office staff room, or cubicle available.

Each EP will then have to present the patients he is actually taking care of, with brief description of the case including the following items:

- Sex, age, chief complaint and main medical history
- Main clinical findings
- Main investigation (laboratory and imaging) results available/outstanding
- Treatment given in the ED
- Brief summary of the plan (suspected diagnosis, discharge/admission)

The CRT will pass a written copy of this plan of cross checking to each EP. After a case has been presented by the EP, the comments and advice of the crosschecker will be sought. Examples of Cross Checking sessions are provided in Table 2

**Table 2:** Examples of cross checking

*Example 1:*
*EP1: I am seeing a 32 year old male, with acute flank pain and history of renal colic.*
*No fever and no guarding, right lumbar excruciating pain. Urinary dipstick is positive for blood. He’s been pain free since we gave him Ibuprofen. I am awaiting his electrolytes results and if normal, will discharge him with outpatient CT scan in the next week and analgesia.*
*EP2: OK. Next patient?*
*Example 2:*
*EP 1: I am seeing a 65 woman with shortness of breath and history of COPD, it is likely Exacerbation of COPD. PH is normal, bicarb 30, no elevated lactate. If the chest X-ray is normal then I will admit her to the ward for a course of nebulizers as she is still dyspneic.*
*EP2 : What are her clinical findings. Have you started any treatment yet?*
*EP 1: She is 140/58, pulse 101, temperature 38.2 °C. I have her on Salbutamol nebulizer.*
*EP 2: Ok. Shouldn’t you consider starting an antibiotics course and prednisolone?*

### Chart review and adjudication of endpoints

First, the local investigator in each center will review charts from his or her center, as detailed below. The local investigator will undergo formal training by the study coordinator including a 60 min training session by telephone; live slides presentation; and practice chart reviews with feedback. This method of training has been used in a previous study by Camargo et al. and is detailed in his previous publications [4, 17]. For all selected patients, a CRT will retrieve the complete medical chart pertaining to the ED visit, and if the patient was admitted into hospital, discharge summaries following hospital discharge. Repeat attendance in the ED within the next 7 days will be recorded. All charts will be blinded to date, period and group. Chart review for SME will then be assessed in a validated two phase review process [3, 4, 17–21] (Fig. 1).

This first chart review phase will use a an adapted validated questionnaire, derived from the NEDSS study [17], as a screen to detect adverse events and near misses (Additional file 1). Any chart that screens positive for at least one item at the first review phase will be sent for external validation and confirmation in the second review phase. These screen-positive charts will be centralized at the methodology and research department and will be independently reviewed by two physicians from a review expert panel in the second chart review phase. This panel will include board-certified emergency physicians and experts in patient safety. Some of the panel members are already trained to chart abstraction and errors validation and classification [11]. The others from the panel will complete a specific training session, with practice chart review and presentation of classification of error in their severity (according to NCCMERP). In cases of disagreement after discussion with the paired reviewer and failure to reach consensus, a third expert, faculty member emergency physician, will be sought to make a final decision. The preventability of any potential adverse event will be reported on a Likert scale as follows: 0) highly unlikely 1) unlikely 2) likely 3) highly likely.

This two-level reviewing system has been widely used in previous studies on medical errors [3, 18–23], although rarely in the ED setting [17]. To evaluate whether the first level is reliable, we will randomly select 100 charts that were initially screened negative on the first level, and send them for external reviewing. If the rate of SME is higher than 2 % (i.e. upper 95 % confidence interval (CI) bound > 5 %), all charts will undergo the second level of reviewing to limit selection bias.

### Statistical analysis

Patient characteristics will be reported in each period, and we will calculate number (rate), mean (standard deviation) or median (interquartile range) when appropriate. Normality will be tested with Kolmogorov-Smirnov method. Proportion of SME will be expressed as percentage and its exact 95 % CI. Characteristics of the two periods will be compared, and differences in any of the following variables will be sought: characteristics of patients and physicians, daily census and severity of patients (triage level, admission rate and ICU admission rate).

The effect of cross-checking will be estimated through a generalized estimating equation (GEE) model, which will take into account the independence of intracluster observations. Factors associated with SME will also be sought with a GEE Model. Severity of SME will be described and compared between the two periods with a chi square test or Fisher exact test when appropriate.

Based on previous literature, we estimate a rate of SME of 10 %, with a potential avoidance rate of more than 50 % [4, 11, 24]. With a hypothesis of a 40 % reduction in the rate of SME (10 % control vs 6 % cross checking), with alpha = 0.05 and beta = 0.2 and accounting for the fact that the cross-over will counterbalance the cluster’s inflation factor, we need to analyze 1584 charts – 140 per period in each center.

All statistical tests will be two-tailed, and a p less than 0.05 will be required to reject the null hypothesis.

## Discussion

Medical errors are common in the ED, with a high rate of adverse events. In 2013, a systematic review reported a substantial variation in the proportion of patients that experienced AE from ED care, ranging from 0.2 to 6 % [24]. Since this review, two other studies confirmed that the actual rate of AE in the ED may vary from 5 to 10 % [4, 11]. Of note, more than 50 % of them are preventable, highlighting the importance of intervention to reduce this rate. The CHARMED study will be the first intervention study that aims to reduce the proportion of patients that experience adverse events from a medical errors in the ED. Studies reporting lower rates were those without systematic reviewing of charts to detect endpoints, but rather a declarative or a passive system, whereas the highest rate of AE (6–10 %) were reported in those that employed the systematic two levels reviewing methodology [4, 11, 25] to detect adverse events. Thus, we belive that our chosen methodology for chart review is the gold standard for the detection of adverse events and seems adapted in the settings of ED.

In other settings, simple interventions, such as the implementation of checklists [26–29] , have been reported to significantly reduce the rate of adverse events. These kind of interventions intended to reduce errors are difficult to implement in the ED setting, considering the broad variety of patients and medical problems. Although handover might be a source of loss of information and medical errors [30], protocolized handover can reduce the rate of AE [18, 23]. Handover can provide an EP with an opportunity to consider their patient’s care with fresh eyes, and may therefore constitute a barrier to human errors. As suggested by Kajdacsy-Balla Amaral et al., a new physician (in the case of this trial, the crosschecker) may be more prone to reevaluate a patient and its management. This hypothesis was expressed in 1982, when Cooper et al. reported that adverse events occurred less when a relieving anesthetist was involved [31]. This is also in line with the results of our pilot study in which the involvement of a second physician (a resident, or a peer for handover [11]) was the only protector from AE due to medical error in the ED. The risk-benefit balance between the risk of missed information, and the advantage of a second opinion may warrant the involvement of a second physician, hence our intention to introduce systematic cross checks.

### Limitations

There is a potential bias of classification in the adjudication of the primary endpoint. Although the method we will employ to detect and validate AE has already been described, our study may underestimate the incidence of SME. The inherent limitations of chart review is a potential cause of bias, but we are not aware of any other better method.

A Hawthorne effect might occur in the intervention period. Being approached every 3 hours for crosschecking, in the presence of a CRT, may increase the EPs’ awareness of the risk of medical errors and AEs. Crosschecking will not be the only factor that influences EPs in their ED care: being observed and analyzed on their risk of errors, EPs could be more careful, and less prompt to error. To reduce this effect, we will advertise the study in the six participating centers, and a CRT will still be present during the control period to remind emergency physicians that a prospective study on medical errors is taking place. Consequently, we will reduce bias by creating the circumstances that predispose to a Hawthorne effect in both periods of the study.

There is a possibility of contamination bias between the two periods, especially in the centers randomized to have the intervention period before the control period: EPs that found the concept of cross checking useful may pursue its application in daily care. To limit this bias, there will be a 4-week wash out interval between the two periods.

Finally, our study will take place in six urban, academic adults ED. For this reason, we cannot generalize to other settings, especially small rural, or pediatric EDs.

The CHARMED study, a randomized cluster cross-over study, will evaluate the efficacy of the implementation of systematic crosschecking, in reducing the rate of severe medical errors in the ED. CHARMED will be the largest study including unselected ED charts reviewed for detection of adverse events.

## Additional file

Additional file 1:
**Adverse Event Form.** (DOC 37 kb)
