# Supplementary material for: Cross-checking to reduce adverse events resulting from medical errors in the emergency department: study protocol of the CHARMED cluster randomized study
Source: BMC Emerg Med. 2015 Sep 4;15:21. doi: 10.1186/s12873-015-0046-1 (PMC4560890; doi:10.1186/s12873-015-0046-1)
Supplement: Additional file 1: — Adverse Event Form. (DOC 37 kb) [file 12873_2015_46_MOESM1_ESM.doc]

**Additional file 1:** Adverse Event Form

**Center :** **PSL** **SAT** **TNN** **AVC** **GRN**

**Patient number:**

**Severe outcome within 7 days**

Death or cardiac arrest Yes No

ICU admission Yes No

Return visit to the ED or re hospitalisation Yes No

Adverse drug event Yes No

Thrombo-embolic event Yes No

Severe hemorrage Yes No

Severe sepsis or septic shock Yes No

Acute coronary syndrome Yes No

One episode of hypotension (SBP<90 mmHg) Yes No

Antidote regimen started (Flumazenil, Prothrombin complex…) Yes No

Other undesirable outcome Yes No

Detail : ………………………………………………………………………………………

**Known at risk situation**

Major procedure (Sedation, intubation, ponction) Yes No

Catecholamine or vasopressor introduction Yes No

Mechanical ventilation or NIV Yes No

Total length of stay in the ED > 4 hours Yes No

Electrolyte disturbances (Dysnatraemia, Dyskalaemia,…) Yes No

**At least one aspect of the ED care is suboptimal**

Yes No

**Suspicion of :**

Guideline violation (local or national) Yes No

Medication error Yes No

Delay for recommended treatment Yes No

Insufficient monitoring Yes No

Error in ordering test Yes No

Delay in ordering test Yes No

Lack of prevention (eg no LMWH) Yes No
